# Pharmacokinetics and Perceptions of Children and Young Adults Using Cannabis for Attention-Deficit/Hyperactivity Disorder and Oppositional Defiant Disorder: Protocol for a Mixed Methods Proof-of-Concept Study

**DOI:** 10.2196/31281

**Published:** 2021-10-18

**Authors:** Holly Mansell, Declan Quinn, Lauren E Kelly, Michael Szafron, Jane Alcorn

**Affiliations:** 1 College of Pharmacy and Nutrition University of Saskatchewan Saskatoon, SK Canada; 2 Division of Child and Adolescent Psychiatry College of Medicine University of Saskatchewan Saskatoon, SK Canada; 3 Department of Pediatrics and Child Health and Community Health Sciences University of Manitoba Winnipeg, MB Canada; 4 School of Public Health University of Saskatchewan Saskatoon, SK Canada

**Keywords:** attention-deficit/hyperactivity disorder, ADHD, oppositional defiant disorder, cannabis, cannabidiol, young adults, youths, pharmacokinetics, marijuana

## Abstract

**Background:**

Despite the lack of evidence on the use of cannabis for the treatment of attention-deficit/hyperactivity disorder (ADHD), the growing perception that cannabis is safe has led more patients and caregivers to self-medicate. Some psychiatrists now authorize medicinal cannabis for patients with ADHD with features of oppositional defiant disorder (ODD) to curtail the unregulated (ie, self-medicated) use of recreational cannabis or to offer a therapeutic option to those who continue to experience symptoms after exhausting all other treatment options.

**Objective:**

This protocol aims to explore the perceived effectiveness and pharmacokinetics of cannabis in youth and young adults, who are currently taking it as part of their treatment plan for ADHD with features of ODD, under the supervision of a psychiatrist.

**Methods:**

Patients between the ages of 12 and 25 years with a diagnosis of ADHD and features of ODD, who are currently taking cannabis herbal extract (at a Δ^9^-tetrahydrocannabinol [THC]:cannabidiol [CBD] ratio of 1:20) as a treatment adjunct to stimulant pharmacotherapy will be recruited. A sample size of 10-20 individuals is estimated. The study interview will consist of (1) validated symptom rating scales (Swanson, Nolan, and Pelham-IV Questionnaire [SNAP-IV], 90-item; Patient Health Questionnaire, 9-item [PHQ-9]; and Screen for Child Anxiety Related Emotional Disorders [SCARED] tool to measure symptoms of ADHD and ODD, depression, and anxiety, respectively); (2) a semistructured interview to probe the experiences of using cannabis; and (3) a cannabis side effects survey. A cannabis product sample as well as 2 blood samples (a trough level and 2-hour postdose level) will be collected to measure plasma concentrations of cannabinoids and relevant metabolites (THC, CBD, 11-hydroxy-THC, 7-hydroxy-CBD, cannabichromene, and 11-nor-9-carboxy-THB) using liquid chromatography–tandem mass spectrometry (LC–MS/MS). Self-report rating scales (SNAP-IV, SCARED, and PHQ-9) will be scored in accordance with standard protocols and compared to retrospective scores obtained from the participant’s chart. Demographic variables (age, weight, and race), symptom scores, and blood levels (peaks and troughs) of THC, CBD, cannabichromene (CBC), and metabolites will be summarized using descriptive statistics. Relationships between plasma concentrations and symptom scores will be determined using analysis of variance, and multiple regression analysis will be performed to determine associations between plasma concentrations and demographic variables (age, weight, and ethnicity). The qualitative data will be audio-recorded and transcribed and organized into themes.

**Results:**

The protocol was approved by the Biomedical Research Ethics Board at the University of Saskatchewan (protocol #1726), and recruitment began in May 2021.

**Conclusions:**

This proof-of-concept study will explore the potential treatment effectiveness of medical cannabis in participants with ADHD and ODD through a mixed methods approach to inform future research in this area.

**International Registered Report Identifier (IRRID):**

DERR1-10.2196/31281

## Introduction

### Background

Attention-deficit/hyperactivity disorder (ADHD) is one of the most common mental health conditions in children, with an estimated worldwide prevalence of 7.2% [1]. This chronic neurobehavioral disorder is characterized by inattention and hyperactivity-impulsivity and affects both children and teens, with up to 60% of those affected exhibiting symptoms into adulthood [2]. Treatment of ADHD typically involves nonpharmacologic strategies (eg, healthy diet, education, and cognitive and behavioral interventions), and pharmacologic therapy with a psychostimulant (eg, methylphenidate) [3]. While stimulant pharmacotherapy is effective for treating the core symptoms of ADHD, in approximately 70%-90% of cases, symptoms of aggression are less likely to respond. Approximately 35%-65% of children with ADHD exhibit comorbid disruptive behavior disorders (DBDs; oppositional defiant disorder [ODD], or conduct disorder) [4,5], and a substantial number continue to exhibit aggressive and disruptive behaviors even after stimulant treatment [4-6]. The consequences of inadequately treated aggressive and disruptive behaviors are significant; these children are more likely to have encounters with the justice system, deficits in academic achievement, behavioral and disciplinary problems, and substance use challenges [7].

### Cannabis Use and ADHD

The growing perception that cannabis may be useful for alleviating ADHD symptoms has motivated individuals to use cannabis without the necessary evidence to support its use and without clear guidance on appropriate dosing [8,9]. A recent study of internet-based discussions about the effects of cannabis on ADHD found at least 3 times as many comments advocating for cannabis’ therapeutic benefits, compared to comments regarding harm or lack of efficacy [10]. Moreover, several parents of children with ADHD have admitted to administering cannabis to their children for symptom management [8,11].

Some adults with ADHD have reported benefits from using cannabis. These benefits include feeling calmer, improved sleep, and the ability to sustain focus [12]. Patients with ADHD typically use cannabis “to improve their mood and sleep” rather than “to get high” [13]. Cannabinoids act on the endocannabinoid system, which is a signaling system consisting of 2 receptor subtypes (CB1 and CB2). ADHD involves a dysregulation of dopamine, and stimulant pharmacotherapy works by blocking the reuptake of dopamine as a result of the inhibition of noradrenergic areas in the prefrontal cortex [14]. CB1 receptors also interact with the dopaminergic system, and it is hypothesized that the modulation of endocannabinoids in the medial prefrontal cortex and the ventral tegmental area may lead to regulation of the impulsive action and restraint [15,16]. Several other neurotransmitters, such as glutamate, γ-aminobutyric acid, and *N*-methyl-D-aspartate, as well as CB2 receptors can interact with endocannabinoids and may contribute to the modulation of impulsivity [15-17].

Co-occurring substance use is one of the most common problems associated with ADHD. Children with ADHD are at an increased risk for both using cannabis and having a cannabis use disorder, and these youth are nearly 3 times more likely to report cannabis later in life compared to the general population [10,18]. Whether or not potential harms associated with substance use are worse for youth with ADHD is currently unknown.

The self-medication theory is one possible theory to explain the increased risk of substance misuse in some patients [19,20]. This hypothesis, which is a theory about addiction, originally focused on why and how individuals were drawn to heroin and cocaine [19]. In 1997, it was updated to consider a variety of other applications [20]. Based on decades of clinical observation, it proposes that patients consume drugs in an effort to cope with the illness or treatment side effects [19,20]. In the case of ADHD, individuals may self-medicate to alleviate negative emotionality, such as anger, sadness, anxiety, and inadequate emotional regulation [21]. In medicine, the decision to initiate a medication or other treatment is based on the theoretical gains in therapeutic benefits weighed against the potential harms [22]. Observational studies indicate that cannabis may improve symptom management and decrease side effects associated with prescription medication, or as a substitute for alcohol and illicit drugs [16,23,24]. Nonmedical cannabis use has also helped to decrease cocaine dependence in a study of patients with ADHD [25]. In this capacity, cannabis substitution could be considered a harm reduction strategy [22]. Some psychiatrists within our institution have now resorted to prescribing medicinal cannabis for patients with ADHD and ODD who were using unregulated cannabis recreationally, or “self-medicating,” or among those who continue to experience symptoms after exhausting all other treatment options. The dearth of evidence regarding cannabis use for ADHD bespeaks an urgent need to determine whether cannabis use is safe and effective in these individuals.

### Goal of the Study

The goal of this pilot study is to examine the real-world effectiveness by comparing changes in their disease-related symptoms before and after beginning treatment with medical cannabis, using validated assessment scales for ADHD, ODD, depression, and anxiety. We will characterize their experiences with using medical cannabis by way of a semistructured interview and cannabis side effects survey. Furthermore, blood levels of commonly found cannabinoids in cannabis in children and young adults who are currently taking medical cannabis for the treatment of their ADHD and ODD under the care of a pediatric psychiatrist will be correlated with symptom scores and demographic variables (age, weight, and ethnicity). Finally, a sample of the participant’s cannabis will be analyzed through liquid chromatography–tandem mass spectrometry (LC–MS/MS) to confirm its chemical composition.

#### Specific Objectives and Hypotheses

Hypothesis 1: Patients who use medicinal cannabis perceive improvements in ADHD and ODD symptoms.

Hypothesis 2: Improvements in self-reported symptom scores associate with higher plasma levels of cannabidiol (CBD) and Δ^9^-tetrahydrocannabinol (THC) and their bioactive metabolites.

#### Primary Objectives

1. Examine the changes between self-report ADHD scores (as measured by the symptom scores of the Swanson, Nolan, and Pelham-IV Questionnaire [SNAP-IV, 90-item]) before (retrospectively) and after the initiation of medical cannabis.
2. Examine potential associations between steady-state plasma concentrations of CBD, THC, cannabichromene (CBC), and well-known metabolites in children with symptom scores of the SNAP-IV (90-item).
3. Characterize the experiences of participants using medical cannabis.

#### Secondary Objective

1. Examine potential associations between self-reported symptom scores from other validated ADHD assessment tools (Screen for Child Anxiety Related Emotional Disorders [SCARED] rating scale, and Patient Health Questionnaire, 9-item [PHQ-9]), and steady-state plasma concentrations of CBD, THC, CBC, and well-known metabolites in children using cannabis for ADHD with ODD.

## Methods

### Study Design and Inclusion Criteria

An observational, mixed methods, proof-of-concept study will be undertaken at 1 center in Saskatchewan. The protocol was designed to minimize face-to-face contact, so the study can be performed during the COVID-19 pandemic. Patients with ADHD and ODD who are currently taking cannabis herbal extract (at a THC:CBD ratio of 1:20) as a treatment adjunct to stimulant pharmacotherapy are eligible to participate. Participants are between the ages of 12 and 25 years; have a diagnosis of ADHD in accordance with Diagnostic and Statistical Manual of Mental Disorders (5th edition) with features of ODD; are stabilized on medical cannabis herbal extract (at a THC:CBD ratio of 1:20); and have been deemed safe to participate by the study physician. Participants under the age of 18 years must also have the permission of a guardian to participate. We acknowledge that some patients with ADHD may have comorbid mental health disorders, such as autism spectrum disorder. For the purpose of this study, though, we will only enroll participants who are functionally able and willing to provide assent. We will aim to enroll at between 10 and 20 participants in this pilot study.

### Enrollment and Consent

Potential participants and their caregivers will be identified and initially contacted by the study physician (DQ) through his childhood and adolescent psychiatry practice at the Saskatchewan Health Authority (SHA) in Saskatoon (Saskatchewan, Canada). If the family is interested in learning more, their contact information will be forwarded to a research team member who will follow up with the family. Potential participants (and guardians, if applicable) who express interest will be provided with a copy of the consent form and the study information reviewed. If the participant (and caregiver, if applicable) opts to participate, logistics will be arranged and informed consent taken.

### Study Interview

#### Overview

A study interview will be performed at a mutually convenient time for the participant (and caregiver, if applicable) and research team member. The interviews are expected to last approximately 40-60 minutes each and will be conducted via Cisco WebEx or phone, depending on the participant’s preference. If the participant requires a break during the interview, we will accommodate this need. The interviews will be audio-taped, and notes recorded by the researcher, but all information gathered from the participant will be kept confidential. Demographic information is collected, including cannabis product and dosing regimen, age, sex, clinical diagnosis, ethnicity, other medications, and participants’ self-reported height and weight. Self-reported rating scales are used to measure participants’ current symptoms of ADHD and debility, and a semistructured interview will be used to explore their experiences of cannabis use.

#### Self-reported Symptom Rating Scales

The SNAP-IV (90-item) is a revision of the original SNAP questionnaire [26,27], which contains items from the Diagnostic and Statistical Manual of Mental Disorders (DSM-IV) criteria to assess inattention (items 1-9), hyperactivity/impulsivity (items 11-19), and ODD (items 21-28). Items have also been added to summarize the Inattention, Hyperactivity/Impulsivity, and ODD domains (items 10, 20, 29, and 30), as well as items representing a general index of childhood problems (items 31-90). Each item measures the frequency or severity of a symptom or behavior, on a Likert scale of 0-3 (0=not at all and 3=very much). This instrument has been shown to have good reliability and validity in different study samples [28].

The PHQ-9 is a 9-item tool used for screening, diagnosing, monitoring, and measuring the severity of depression [29] (Multimedia Appendix 1). Each item is scored on a Likert scale of 0-3 (0=not at all and 3=nearly every day). The items are summed to equal a total between 0 and 27, with higher scores equating to a higher level of debility [29].

The SCARED tool, which assesses anxiety symptoms, consists of 41 items and 5 factors that parallel the DSM-IV classification of anxiety disorders [30]. Each item is scored on a Likert scale of 0-2 (0=not true or hardly ever true and 2=very true or often true). A score of ≥25 may indicate the presence of an anxiety disorder and scores higher than 30 are increasingly specific [30].

#### Cannabis Use Questions

A semistructured interview guide will be used to characterize the perceptions of participants (and guardians, if applicable) of cannabis treatment. The interview guide was drafted a priori by the research team and was piloted on 3 patients who use cannabis therapeutically. The interview consists of 6 open-ended questions, which explore participant’s life circumstances before initiating medical cannabis, contributing factors for choosing this treatment*,* how (if at all) things have changed, what concerns (if any) might exist about treatment, and how the participant obtains the medical cannabis (Multimedia Appendix 2)*.* The interviews will be flexible, depending on the participant’s responses and probes will be used to delve further into potential areas of interest. No time restrictions will be placed on the interview. Rather, the conversation continues until data saturation is reached, and no further information is offered from the participant. Field notes will be taken throughout the interview to capture nuances of the conversation. Finally, a cannabis side effect survey [31] will be administered, which capture potential side effects experienced from taking cannabis within the previous week. Potential side effects in this survey are categorized under the domains of cognitive, physiological, psychological, movement, and artistic/social, and response choices for each item include “yes,” “no,” or “uncertain.”

#### Blood Collection

Within 1 week of the interview, the mobile laboratory will visit the participant’s residence to obtain 2 blood samples for evaluation of the plasma levels of CBD, THC, CBC, and active metabolites. Measured metabolites will include 11-hydroxy-Δ^9^-THC (11-OH-THC) and 7-OH-cannabidiol. A trough level (immediately before the morning cannabis dose) will be collected to represent the minimum steady-state plasma drug concentration (C_SS,min_), while a 2-hour postdose level is collected to represent the maximum steady-state plasma drug concentration (C_ss,max_; where therapeutic effect should be the highest) [32,33]. Blood samples (1 mL each) will be collected into BD Vacutainer Barricor tubes [34] and centrifuged at 2000 × g for 5 minutes to separate plasma. Samples will be subsequently transferred to Eppendorf Protein LoBind microcentrifuge tubes and transported on ice, until they reach the laboratory for storage in a –80°C freezer.

Concurrent medications will be continued by the participant as per usual. No dietary restrictions are imposed on the day of the pharmacokinetic analysis, to capture the real-world situation of patients using cannabis herbal extract as an adjunct treatment to stimulant therapy.

#### Cannabis Sample Collection

Participants are provided with an option to have a small sample (<0.5 mL) of their cannabidiol oil collected on the day of the blood collection, to be analyzed in the laboratory. The purpose is to confirm the composition of the cannabis product. The results of this analysis will be communicated back to the participant.

### Data Analysis

#### Sample Size Determination and Power Calculation

Since there is an absence of literature in this area, with this proof-of-concept study, we aim to recruit as many subjects as possible up to a maximum of 20 participants. The data obtained from this pilot analysis will be used to inform future clinical studies.

#### Cannabis Analysis

The medical cannabis product sample will be analyzed for the major cannabinoids in the product (eg, THC, CBD, and CBC). The plasma concentrations obtained from the participant will undergo analysis for the major cannabinoids and their relevant metabolites (THC, CBD, 11-OH-THC, 7-OH-CBD, CBC, and 11-nor-9-carboxy-THC (THC-COOH) using LC–MS/MS. This method has been previously developed and validated within our institution [35] in accordance with the guidelines of the Food and Drug Administration [36].

#### Quantitative Analysis

The self-reported rating scales (SNAP-IV, SCARED, and PHQ-9) will be scored in accordance with standard guidelines. Changes in rating scores will be determined by subtracting the baseline score (obtained prior to cannabis initiation) available in the participant’s medical chart, from the final score obtained during the interview. Demographic variables (age, weight, and racial background), symptoms scores, and blood levels (peaks and troughs) of THC, CBD, CBC, and metabolites will be summarized using descriptive statistics. Adverse effects will be summarized descriptively or listed. Differences between plasma concentrations and symptom scores will be determined using analysis of variance (ANOVA) and multiple regression analysis will be used to determine associations between plasma concentrations and demographic variables (age, weight, and racial background). Spearman ρ will be used to calculate correlation coefficients. To control for the increased type I error resulting from these multiple comparisons, the level of significance will be set to *P*≤.01. Statistical analyses will be performed using SPSS (version 27, IBM Corp).

#### Qualitative Analysis

Audiotapes from the interviews will be transcribed verbatim and the data will be input into NVivo qualitative software. The data will be coded by one of the study investigators and will be reviewed by one of the primary investigators. Discrepancies between the investigators will be resolved through discussion and debate and the second primary investigator will weigh in if needed. The data will be organized into common themes and summarized.

## Results

The protocol was approved by the Biomedical Research Ethics Boards at the University of Saskatchewan (protocol #1726). Recruitment began in May 2021.

## Discussion

### Principal Findings

Legalization of recreational cannabis occurred in Canada in October 2018. The increased public accessibility, coupled with perceptions that cannabis is “natural” and perhaps “safer” than some of the other available pharmacotherapeutic agents [8,9], has increased the probability of cannabis use in this population, despite an absence of evidence on efficacy or safety in youth or young adults.

At the time this study was designed, only 1 controlled clinical trial was published on the use of cannabis in ADHD. Cooper et al [37] performed a (pilot) randomized controlled trial using Sativex Oromucosal Spray (1:1 THC:CBD) or placebo, in 30 adults with ADHD. Participants in the Sativex group demonstrated a pattern of improved cognitive performance as measured by the QbTest, nominally significant improvements in symptoms of hyperactivity/impulsivity (*P*=.03), and trends toward improvement for inattention and emotional lability. All trends were strengthened when the per-protocol analysis was performed [37]. Case reports have also described the beneficial effects of cannabis on ADHD symptoms [16,38].

Controlled clinical trials are clearly needed to determine the impact of cannabis use on ADHD symptoms in youth and young adults. Understanding the impact of pharmacotherapy is of particular importance in the pediatric population, where development may be adversely and unpredictably impacted by drug therapy [39]. However, pilot studies need to precede interventional studies to understand feasibility and to glean important information about the pharmacokinetics of cannabis in the pediatric patient population to guide dosing strategies.

While the small sample size of this pilot study will preclude treatment recommendations, the importance of this study should not be understated. Exploring the real-world effectiveness and pharmacokinetics of cannabis in a cohort that is already taking cannabis is the most ethical way to gather the necessary information for initiating a research program in this area. If the results from this pilot study are positive, our future work will include a single dose pharmacokinetic study and eventually a randomized controlled trial.

### Conclusions

Novel treatment strategies are needed for patients who experience symptoms of ADHD and ODD despite stimulant pharmacotherapy. Some desperate families have resorted to using cannabis, despite the lack of safety or efficacy data. This pilot study will be the first to explore the real-world effectiveness, perceptions, and pharmacokinetics of cannabis in children and young adults, and the results will guide future study in this area.

## Appendix

Multimedia Appendix 1 Questions from the Patient Health Questionnaire, 9-item (PHQ-9).

Multimedia Appendix 2 Semistructured Interview Guide.

## Abbreviations

11-OH-THC: 11-hydroxy-Δ9-tetrahydrocannabinol
ADHD: attention-deficit/hyperactivity disorder
CBC: cannabichromene
CBD: cannabidiol
C_ss,max_: maximum steady-state plasma drug concentration
C_ss,min_: minimum steady-state plasma drug concentration
DSM-IV: Diagnostic and Statistical Manual of Mental Disorders
LC–MS/MS: liquid chromatography–tandem mass spectrometry
ODD: oppositional defiant disorder
PHQ-9: Patient Health Questionnaire, 9-item
SCARED: Screen for Child Anxiety Related Emotional Disorders
SHA: Saskatchewan Health Authority
SNAP-IV: Swanson, Nolan, and Pelham-IV Questionnaire
THC: Δ9-tetrahydrocannabinol
THC-COOH: 11-nor-9-carboxy-tertrahydrocannabinol
