# Supplementary material for: Pharmacokinetics and Perceptions of Children and Young Adults Using Cannabis for Attention-Deficit/Hyperactivity Disorder and Oppositional Defiant Disorder: Protocol for a Mixed Methods Proof-of-Concept Study
Source: JMIR Res Protoc. 2021 Oct 18;10(10):e31281. doi: 10.2196/31281 (PMC8561403; doi:10.2196/31281)
Supplement: Multimedia Appendix 1 [file resprot_v10i10e31281_app1.docx]

Questions from the Patient Health Questionnaire (PHQ-9)

|  | Over the last 2 weeks, how often have you been bothered by any of the following problems? | Not at all | Several days | More than half the days | Nearly every day |
| --- | --- | --- | --- | --- | --- |
| 1 | Little interest or pleasure in doing things | 0 | 1 | 2 | 3 |
| 2 | Feeling down, depressed, or hopeless | 0 | 1 | 2 | 3 |
| 3 | Trouble falling or staying asleep, or sleeping too much | 0 | 1 | 2 | 3 |
| 4 | Feeling tired or having little energy | 0 | 1 | 2 | 3 |
| 5 | Poor appetite or overeating | 0 | 1 | 2 | 3 |
| 6 | Feeling bad about yourself – or that you are a failure or have let yourself or your family down | 0 | 1 | 2 | 3 |
| 7 | Trouble concentrating on things, such as reading the newspaper or watching television | 0 | 1 | 2 | 3 |
| 8 | Moving or speaking so slowly that other people could have noticed? Or the opposite – being so fidgety or restless that you have been moving around a lot more than usual | 0 | 1 | 2 | 3 |
| 9 | Thoughts that you would be better off dead or of hurting yourself in some way | 0 | 1 | 2 | 3 |

Kroenke K, Spitzer RL, Williams JB. The PHQ-9: Validity of a brief depression severity measure. J Gen Intern Med. 2001;16(9):606-613.
