# Supplementary material for: Pharmacokinetics and Perceptions of Children and Young Adults Using Cannabis for Attention-Deficit/Hyperactivity Disorder and Oppositional Defiant Disorder: Protocol for a Mixed Methods Proof-of-Concept Study
Source: JMIR Res Protoc. 2021 Oct 18;10(10):e31281. doi: 10.2196/31281 (PMC8561403; doi:10.2196/31281)
Supplement: Multimedia Appendix 2 [file resprot_v10i10e31281_app2.docx]

Semi-structured interview guide

|  | This set of questions will help us learn more about your experience with *Cannabis*. Please note, you do not have to answer any questions you do not feel comfortable with. |
| --- | --- |
| 1 | Tell me about your life growing up with ADHD prior to taking medical *Cannabis*? |
| 2 | What led you to choose medical *Cannabis/* what was the reason for starting medical *Cannabis*? |
| 3 | How have things changed for you (if at all) since beginning *Cannabis*? |
| 4 | What concerns (if any) might you have about taking medical *Cannabis*? |
| 5 | Tell me how you get the *Cannabis*? |
| 6 | What else do you want me to know about your experience with *Cannabis* or ADHD? |
